# Intracellular growth of *Mycobacterium avium* subspecies and global transcriptional responses in human macrophages after infection

**DOI:** 10.1186/1471-2164-15-58

**Published:** 2014-01-23

**Authors:** Angelika Agdestein, Anya Jones, Arnar Flatberg, Tone B Johansen, Inger Austrheim Heffernan, Berit Djønne, Anthony Bosco, Ingrid Olsen

**Affiliations:** 1 Norwegian Veterinary Institute, PO. Box 750 Sentrum, N-0106 Oslo, Norway; 2 Telethon Institute for Child Health Research, UWA Centre for Child Health Research, University of Western Australia, 100 Roberts Rd, Subiaco, WA 6008 Australia; 3 Department of Cancer Research and Molecular Medicine, NTNU, Trondheim, Norway

**Keywords:** *Mycobacterium avium*, Human macrophages, Gene expression

## Abstract

**Background:**

*Mycobacterium avium* subsp. *avium* (*Maa*) and *M. avium* subsp. *hominissuis* (*Mah*) are environmental mycobacteria and significant opportunistic pathogens. *Mycobacterium avium* infections in humans and pigs are mainly due to *Mah*. It is not known whether this is caused by a difference in virulence or difference in exposure to the two subspecies. The aim of the present study was to investigate the ability of the *M. avium* subspecies to replicate intracellularly and to characterise the gene expression program triggered by infection of human primary macrophages.

**Results:**

All isolates were able to invade and persist within human macrophages. However, intracellular replication was only evident in cells infected with the two *Maa* isolates. Transcriptional responses to the isolates were characterized by upregulation of genes involved in apoptosis, immune- and inflammatory response, signal transduction and NF-kB signaling, cell proliferation and T-cell activation. Although similar pathways and networks were perturbed by the different isolates, the response to the *Maa* subspecies was exaggerated, and there was evidence of increased activation of type I and II interferon signaling pathways.

**Conclusion:**

*Mycobacterium avium* isolates of different genetic characteristics invaded monocytes and induced different degree of macrophage activation. Isolates of *Maa* were able to replicate intracellularly suggesting that differences in exposure, uptake or induction of adaptive immunity are more likely explanations for the difference in prevalence between *M. avium* subspecies.

## Background

The *Mycobacterium avium* complex comprises a group of environmental and opportunistic intracellular pathogens [1] with the ability to persist within macrophages and escaping the host’s killing mechanisms [2]. The complex is constituted by the subspecies of *M. avium* together with *M. intracellulare*[3]. Two subspecies of clinical importance for humans and animals are *M. avium* subsp. *hominissuis* (*Mah*) and *M. avium* subsp*. avium* (*Maa*) [4]. *Mah* is the subspecies mainly isolated from porcine and human cases of MAC infection [4], while *Maa* is the causative agent of avian tuberculosis, but is occasionally responsible for *M. avium* infections in mammals [5]. Little is known about the routes of transmission and the virulence mechanisms of *M. avium*, and a zoonotic potential has not been ruled out [6].

Definite reasons for *Mah* being the dominating subspecies in humans and pigs infected with *M. avium* have not been identified, although differences in exposure could be considered a contributing factor. Explanations to the higher prevalence of *Mah* in the human and porcine population might also be found in differences between *Mah* and *Maa* at the molecular level of interaction between the host and mycobacterium, a process crucial to the outcome of infection, which therefore might affect the prevalence of the subspecies. However, little has been done to investigate whether *Mah* and *Maa* interact differently with their target cells.

Mycobacterial virulence depends on the ability of these bacteria to invade, persist and replicate within the hostile environment in macrophages of the host organism [7]. This is achieved by the protective properties of the mycobacterial cell wall and by interference with the host’s immune response [8,9]. To assess the ability of *Mah* and *Maa* to grow intracellularly, the use of primary cells will give a more representative result compared to immortalised macrophage cell-lines. Tissue resident macrophages represent a heterogeneous population and it is not clear whether *M. avium* infect a particular phenotype. Monocyte derived macrophages matured in absence of any stimuli give rise to a mixed culture of both the M1 and M2 phenotype [10] and such cultures may thus be considered appropriate for infection studies with *M. avium*. Finally, using macrophages derived from the relevant target species is important since the global inflammatory response evoked in mice recently was demonstrated to be vastly different from humans [11]. To the authors’ best knowledge, comparative studies of the ability of *Maa* and *Mah* to invade, replicate and trigger immune responses in human primary macrophages have not been published. The aim of the present study was therefore to assess the growth of *M. avium* isolates in human primary macrophages and to characterise the response triggered in infected macrophages by selected isolates. The isolates used in the present study represented the two different subspecies, were derived from different host species and differed in biofilm abilities, glycopeptidolipid (GPL) genes, and in the presence of the insertion sequence (IS) element IS*Mpa1*[12]. The present data showed that *Maa* replicated to a larger extent in human macrophages suggesting that an inability to grow intracellularly is not likely the cause of the discrepancy of the prevalence of the two subspecies in humans. Furthermore, the response induced in the macrophages could not entirely explain the difference in growth rate.

## Methods

### Mycobacterial isolates

Four clinical isolates of *M. avium* subsp. *hominissuis* (*Mah* VI101, *Mah* 1655, *Mah* H1 and *Mah* H38) and two clinical isolates of *M. avium* subsp. *avium* (*Maa* 1794 and *Maa* 1553) were used in the present study. Details on origin, biofilm producing abilities, presence of IS*Mpa1*, nsGPL- and ssGPL genes were described by Johansen et al. [12], and are summarised in Table 1. The human isolates were from a strain collection at the The Norwegian Institute of Public Health. All isolates were grown in Middlebrook 7H9 (BD Diagnostics, Sparks MD) w/OADC (BD Diagnostics), 1% Tween 80 and 10% glycerol under agitation for two weeks before aliquots were stored at −70°C in an equivalent medium containing 10% glycerol. The concentration of bacteria in the stock suspensions was determined by colony forming unit (CFU) counts. Bacterial inocula for the gene expression assay were prepared from seven days subcultures grown on plates of Middlebrook 7H10 (BD Diagnostics) with 10% OADC (BD Diagnostics) at 37°C. To adjust the number of bacteria in the inoculum, *hsp*65 real-time PCR [13] was performed on serial dilutions in HBSS (Invitrogen, Oslo, Norway) of a suspension adjusted to McFarland standard 2.0, homogenised through a 23 G needle to minimise clumping.

**Table 1:** **Characteristics of *Mycobacterium avium *isolates used in the present study**

**Isolate**	**Subspecies**	**Origin**	**Biofilm**	**IS** ***Mpa1***	**nsGPL**	**ssGPL**
**VI101**	*Hominissuis*	Porcine	+	-	+	-
**1655**	*Hominissuis*	Porcine	-	+	-	-
**H1**	*Hominissuis*	Human	-	-	+	-
**H38**	*Hominissuis*	Human	-	-	+	+
**1553**	*Avium*	Avian	-	-	+	+
**1794**	*Avium*	Avian	-	-	+	+

Isolates of *Mycobacterium avium* used in the present study show different combinations of subspecies designation, origin, biofilm abilities and the genomic presence of IS*Mpa*1 and genes involved in the synthesis of non-specific (ns) or serovar specific (ss) GPLs [12].

### Cell culture

Peripheral blood mononuclear cells (PBMCs) were isolated from buffy coat drawn from healthy human blood donors. The material was commercially obtained from a blood donation centre, and the study was approved by the Regional Committee for Medical Research Ethics, South-East Norway. Fifty mL buffy coat was diluted 1:1 with RPMI 1640 (Sigma-Aldrich, Oslo, Norway) with 1% L-glutamine (Sigma-Aldrich). PBMCs were harvested by density gradient centrifugation with Lymphoprep (Medinor, Bryn, Norway) and resuspended in PBS with 0.5% BSA and 2 mM EDTA at pH 7.2. Isolation of CD14+ cells was performed using CD14 MACS magnetic beads (Milteny Biotec, Bergisch Gladbach, Germany) according to the manufacturer’s instructions. The cells were resuspended in RPMI 1640 with 1% L-glutamine supplemented with 10% human serum to the desired concentration of the respective infection assay, added to the appropriate Costar® cell culture plates (Sigma-Aldrich) and incubated at 37°C with 5% CO_2_. The CD14+ cells were incubated overnight before used in the uptake and replication assays and allowed to mature into macrophages by incubation for five days before used in the gene expression study.

### Uptake and intracellular replication

Uptake and intracellular replication of the six *M. avium* isolates in human blood derived CD14+ cells were assessed. The assays were performed in 96-well cell culture plates (4 × 10^5^ cells/mL, 125 μl per well), which in our hands gave a stable monolayer for most donors. Each isolate was tested in triplicates at an MOI of 5:1. The cells were washed twice with pre-warmed medium to remove extracellular bacteria before the quantification of intracellular bacteria was performed. Intracellular uptake of the bacterial isolates was assayed after three, six, 12 and 24 hours in cells from three donors.

Intracellular replication of the same isolates was assayed in six other donors, following the protocol described above. Based on the results from the uptake studies, CD14+ cells were incubated with the bacterial isolates for six hours. After removal of extracellular bacteria, fresh medium was added and the cells were incubated further. Cell lysates were harvested at six hours, and after one, four and seven days of infection.

### Quantitation of bacteria and cells

Numbers of intracellular bacteria and cells was determined by enumeration of mycobacterial and human genomes in cell lysates. Replication of bacterial isolates in the supernatant had been tested and found insignificant. Lysis of cells was achieved through adding milliQ water to the wells. After 30 minutes, lysates were transferred into 2 mL O-ring vials (Biospec products, Techtumlab, Umeå, Sweden) containing 200 μL silica beads (Biospec products) and stored at −20°C. Lysates from triplicate wells were pooled. After completing all sampling, lysates were thawed and inactivated at 100°C for 30 min, prior to mechanical disruption using the MiniBeadBeater (Techtumlab) for 45 sec.

Enumeration of mycobacteria and cells was achieved by performing a duplex real time PCR of a 103 bp long segment of the mycobacterial *hsp65* gene and a 150 bp long segment of the human β-globin gene on the lysates, as described by Salte et al. [13]. Briefly, the segment of the mycobacterial gene *hsp65* (*GroEl2*) was amplified using the primers MycoFP1 (5′CGAGGCGATGGACAAGGT-3′) and TB 12 (5′CTTGTCGAACCGCATACCCT-3′), and the VIC tagged TaqMan-MGB probe MycoPr1 (5′-AACGAGGGCGTCACCGTCG-3′) [14]. The segment of the human β-globin gene was amplified by using the primers BG-F (5′-TGCCTATCAGAAAGTGGTGGCT-3′) and BG-R (5′-GCTCAAGGCCCTTCATAATATCC-3′), and the FAM tagged TaqMan-MGB probe BG-TAQ (5′-TGGCTAATGCCCTGGCCCACAA-3′) [13,15]. Dilutions of known concentrations of commercially available mycobacterial DNA (ATCC-19015D-5, LGC Standards, Middlesex, UK) and human DNA (Applied Biosystems, Foster City, CA, USA) were run together with the samples and used to create standard curves. The conversion of the known amount of standard DNA to number of bacterial and human genomes were described by Salte et al. [13], as well as the validation of the number of mycobacterial targets against CFU counts. Total volume of each reaction mixture was 20 μl, consisting of 8 μl lysate, 10 μl Quantitect® mastermix (Quiagen, West Sussex, UK) and 1 μl of a primer/probe mix for each target, with a stock concentration of 8 μM for each primer and 4 μM for the probe, resulting in a final primer- and probe concentration of 0.4 μM and 0.2 μM, respectively. Samples and standard DNA dilutions were run on a Stratagene Mx3005P MxPro v 4.10 (Stratagene, La Jolla, CA, USA) and data analysed by the Stratagene MxPro v 4.10 software (Stratagene) with the following thermal cycling conditions: 95°C for 15 min, followed by 40 cycles of 95°C for 1 min and 60°C for 1 min.

### Gene expression profiling

Isolates *Mah* VI101, *Mah* 1655 and *Maa* 1794, were used to infect macrophages for gene expression analysis. CD14+ cells were obtained as described above and allowed to mature into macrophages by incubation for five days in duplicate wells (6 well plates, 1 × 106 cells/mL, 2 ml/well). The cells were then infected with the three isolates at a MOI of 10:1 for four hours. Total RNA was isolated of the adherent macrophages using the RNeasy Mini Kit® (Qiagen, Hilden, Germany), following the manufacturer’s instructions. RNA from the different donors within each assay was pooled per bacterial isolate. The first assay was performed in macrophages from three donors and subsequently repeated twice in two new donors per assay. The quality and integrity of RNA was confirmed by Agilent 2100 Bioanalyzer (Agilent Technologies) and Nanodrop™ (Thermo Fisher Scientific, Wilmington, DE, USA). Gene expression levels were profiled using Illumina HumanHT-12 Expression Bead Chip (Illumina Inc., San Diego, CA, USA). The gene expression data was exported from Illumina’s GenomeStudio software package without background subtraction or normalization. The raw data was preprocessed in the R language and environment for statistical computing [16] employing the normexp by control (neqc) algorithm [17]. Probes that were not significantly expressed above background (detection p-value < 0.01 in at least one sample) were filtered out of the analysis. Differentially expressed genes were identified employing Bayesian/moderated *t*-statistics with False Discovery Rate (FDR) control for multiple testing [18]. Differentially expressed genes were identified at FDR < 0.05. The raw microarray data have been made publicly available in the Array Express repository (http://www.ebi.ac.uk/arrayexpress/experiments/E-MTAB-1101/).

### Bioinformatics

Biological functions enriched in the differentially expressed genes were identified using modular enrichment analysis with DAVID (Database for Annotation, Visualization and Integrated Discovery) [19,20]. Enrichment scores > 1.3 were considered statistically significant. Biological pathways enriched in the data were identified using IPA software (Ingenuity Systems, Redwood City, CA). Upstream regulator analysis was performed in IPA software to infer the putative pathways that give rise to the observed gene expression changes. Molecular interaction networks were reconstructed from the differentially expressed genes in IPA software using mechanistic data from prior studies [21]. In this analysis differentially expressed genes were defined at FDR < 0.01.

### Protein analysis

CD14+ cells were isolated and matured as described above for gene expression studies. Cell lawns were infected at an MOI of 5:1 for 24 hours with *Maa* 1794, *Mah* VI101 and *Mah* 1655. Supernatants were removed for cytokine profiling, and subsequently the adherent cells were washed twice in PBS and collected with a cell scraper in 100 μl of 1x SDS Sample Buffer for immunoblotting. All samples were stored at −70°C until further processing.

### SDS-PAGE and immunoblotting

Proteins were separated by horizontal SDS-PAGE with precast gradient ExcelGel™ SDS Gradient 8 – 18% (GE Healthcare Life Sciences, Uppsala, Sweden) and ExcelGel™ SDS Buffer strips (GE Healthcare) in a Multiphor™ II 2117 (GE Healthcare), prior to diffusion blotting onto nitrocellulose membranes with pore size 0.2 μm (Schleicher & Schuell, Dassel Germany) [22]. Proteins remaining within the gel were visualized by Coomassie brilliant blue staining. Membranes were blocked with PBS with 2% BSA and incubated over night with primary antibodies (Cell Signalling Technology, Danvers, MA) recognizing the following selected proteins involved in apoptosis: Cleaved caspase-3 (Asp175), cleaved caspase-8 (Asp391), cleaved caspase-9 (Asp315), cleaved caspase-9 (Asp330), Bcl-2, Phospho-Bcl-2 (Ser70). Detection of bound antibodies was performed by incubaction with ECL™ Anti-rabbit IgG, Horseradish peroxidise labelled linked F(ab’)2 fragment from donkey (GE Healthcare) and visualization by Immun-Star™ WesternC™ Kit (Bio-Rad, Hercules, CA).

### Cytokine profiling

The concentration of TNF-α, IL-23(p19), IL-10, IL-8 and IL-6 was measured in supernatants by using Bio-Plex™ Cytokine Assay (Bio-Rad) within the Bio-Plex suspension array system (Bio-Rad), following the manufacturer’s instructions. Differences between isolates were tested for significance using the Wilcoxon matched pairs signed-rank test, considering p-values ≤ 0.05 significant.

## Results

### Uptake and intracellular replication

**Figure 1:**
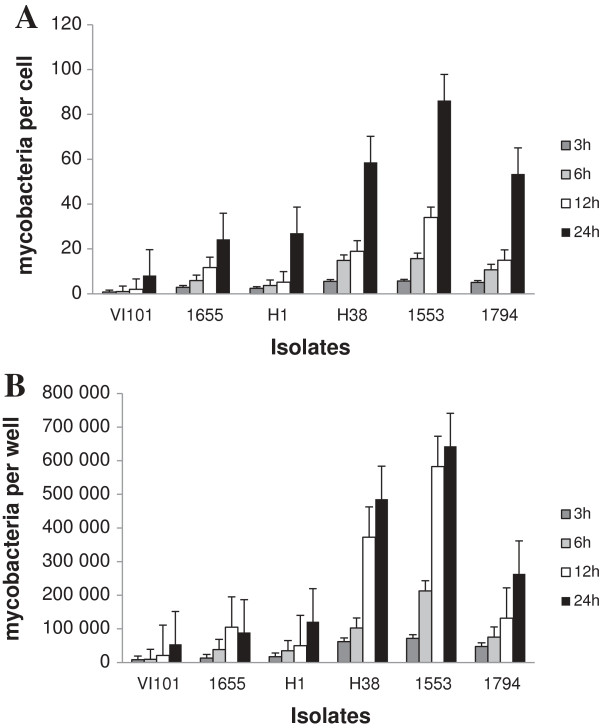
**Uptake of six clinical isolates of *Mycobacterium avium *in human CD14+ cells.** Cells were inoculated for three, six, 12 and 24 hours. Numbers represent the mean values from three donors, showing the ratio of numbers of mycobacterial to human genomes **(A)** and absolute numbers of mycobacteria per well **(B)**, measured by real-time PCR of single copy genes in cell lysates.

All the isolates were taken up by the CD14+ cells, and intracellular bacteria were detected after 3 hours (Figure 1). *Maa 1553, Mah* H38 and *Maa* 1794 appeared to be taken up to a greater extent, while the *Mah* isolate VI101 showed the lowest degree of uptake in macrophages. Between six and 12 hours of infection some of the isolates showed an exponential increase in the number of intracellular bacteria, suggesting the event of onset of intracellular replication. Six hours was therefore chosen as the time between inoculation and removal of extracellular bacteria for the subsequent study of intracellular replication over time. At this point in time VI101 had significantly (p < 0.05) lower numbers of intracellular bacteria compared to the other isolates except for *Mah* H1, while no significant difference were observed between the other isolates using the paired t-test.

**Figure 2:**
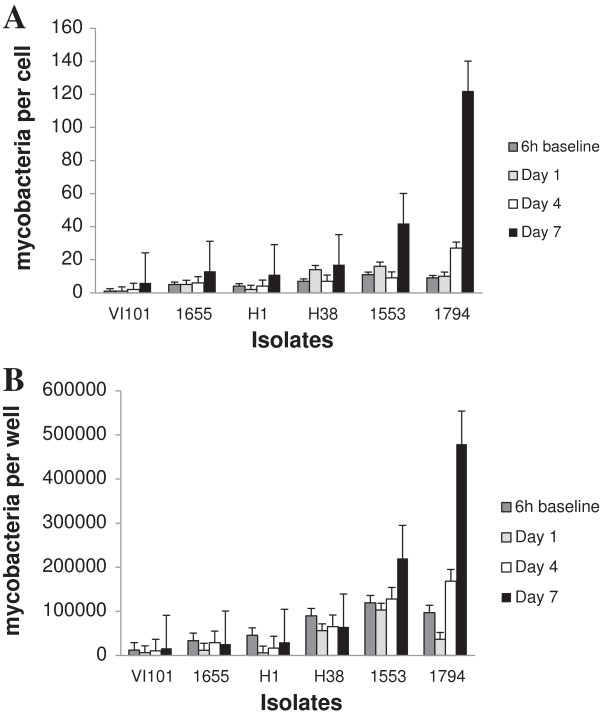
**Intracellular replication of six clinical isolates of *Mycobacterium avium *in human CD14+ cells.** Sampling was performed after six hours of inoculation with added bacteria, then one, four and seven days after removal of extracellular bacteria. Numbers represent the mean values from six donors, showing the ratio of numbers of mycobacterial to human genomes **(A)** and absolute numbers of mycobacteria per well **(B)**, measured by real-time PCR of single copy genes in cell lysates. There was a significant increase in the ratio of mycobacteria at day four for isolate *Maa* 1794 (p = 0.04) and at day 7 for *Maa*1794 (p = 0.01) and *Maa* 1553 (p = 0.02) and in absolute numbers of bacteria for isolate *Maa* 1794 (p = 0.01) at day 7 using the paired t-test.

Intracellular growth was only observed for the two *Maa* isolates. There was a significant increase in the ratio of mycobacteria to human genomes at day four for isolate *Maa* 1794 (p = 0.04) and at day 7 for *Maa* 1794 (p = 0.01) and *Maa* 1553 (p = 0.02) and in absolute numbers of bacteria for isolate *Maa* 1794 (p = 0.01) at day 7 using the paired t-test (Figure 2). The remaining isolates, which were all *Mah*, did not show significant increase in intracellular numbers throughout the duration of infection, however the count of intracellular bacteria was quite stable, indicating that the bacteria were able to persist within the macrophages.

Enumeration of both cells and intracellular mycobacteria was performed by real-time PCR of single-copy genes. This method does not distinguish between viable and non-viable bacteria and cells, but it has the advantage that the number of bacteria is not influenced by the degree of clumping which varies between *Maa* and *Mah*. Furthermore it enabled the measurement of the number of intracellular bacteria per cell in addition to absolute counts of bacteria. Some degree of cell death and loss of adherence were observed in both infected and non-infected cells, and varied considerably between donors. Similar results were observed whether absolute numbers of bacteria or the bacteria per cell ratio were used, however the difference in cell death between donors may explain why the absolute counts of bacteria only reached a significant level of p < 0.05 for *Maa* 1794 at day 7, while the ratio was significant for both *Maa* isolates at day 7 and for *Maa* 1794 at day 4.

### Gene expression analysis and bioinformatics

Based on the results from the initial replication assays, three isolates were selected for gene expression studies in macrophages. *Maa* 1794, which replicated intracellularly, and *Mah* VI101, which only persisted within cells, were selected, in addition to *Mah* 1655, which, as opposed to the other two isolates, contains the IS element IS*Mpa1* and belong to an RFLP type that has been demonstrated to be particularly virulent in pigs [23,24].

**Figure 3:**
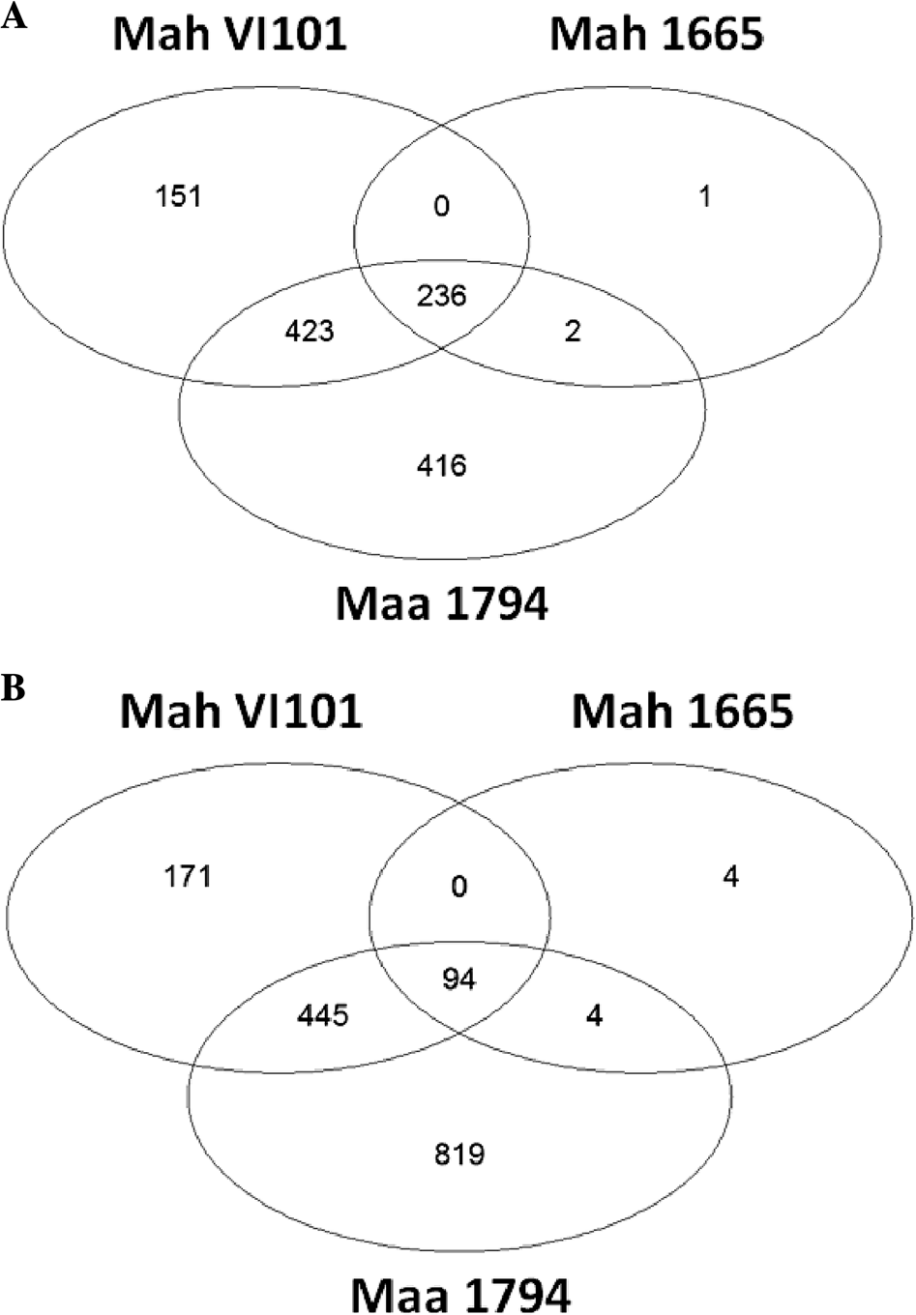
**Numbers of differentially expressed genes in human macrophages infected with different isolates of *Mycobacterium avium*.** The Venn diagrams of numbers of up-regulated **(A)** and down-regulated **(B)** genes show unique and common differential expression of transcripts in human macrophages in response to four hours of incubation by three different isolates of *Mycobacterium avium. Maa* 1794 is an intracellularly replicating isolate of *M. avium* subsp. *avium*, whereas *Mah* VI101 and *Mah* 1655 are isolates of *M. avium* subsp. *hominissuis* that only invade and persist within cells.

The Illumina® HumanHT-12 v3 Expression Bead allows whole-genome expression analysis of 12 samples on the same chip, each array targeting genomewide with more than 48,000 probes. Of the 14,581 probe sets with signal intensity above background, 2,766 probe sets were differentially expressed (FDR < 0.05) in the cells infected with *M. avium* (1,229 up-regulated, 1,537 down-regulated), regardless of the individual isolate, compared to uninfected control cells (Additional file 1: Table S1). The largest response was induced by *Maa* 1794 where 2,439 probe sets (representing 2,147 unique genes) were differentially expressed. Of these probe sets, 1,077 were up-regulated, and 1,362 were down-regulated. *Mah* VI101 modulated expression of 1,520 probe sets (810 up-regulated, 710 down-regulated) representing 1,348 unique genes. In comparison, *Mah* 1665 altered the expression of only 341 probe sets (239 up-regulated 102 down-regulated) representing 298 unique genes. A large overlap was observed in the responses elicited by the three isolates (Figure 3). There were virtually no genes (5 transcripts only) that were uniquely modulated by Isolate *Mah* 1655. In contrast, hundreds of genes were uniquely differentially expressed in response to infection with *Maa* 1794 and *Mah* VI101.

Bioinformatics analyses demonstrated that the major biological functions up-regulated in response to the three isolates were apoptosis, inflammatory response, cytokine/chemokine activity, signal transduction, cell-proliferation and regulation of T-cell activation (Table 2). Biological pathways up-regulated in the responses were related to apoptosis and innate immunity and cytokine signaling (Table 3). In addition, communication pathways between innate and adaptive immune responses and IL-17 signaling were up-regulated. Upstream regulator analysis was performed to infer the putative molecular mechanisms that give rise to the observed gene expression changes, and is illustrated in Table 4. This analysis revealed major roles for TNF, NFkB, IL-1B, IFNg and others in the regulation of the responses. Altogether these analyses revealed a strong overlap between the impacts of all the three isolates.

**Table 2:** **Analysis of biological functions induced infection of human monocyte-derived macrophages with *M. avium *isolates**

	**Biological functions**	**Enrichment score**
*Mah* VI101 compared to uninfected	Negative regulation of apoptosis	15.2
	Inflammatory response	13.5
	Induction of apoptosis	12.7
	Cytokine/chemokine activity	8.4
	Regulation of cytokine production	7.1
	Regulation of signal transduction	6.6
	Regulation of T and B cell activation/proliferation	6.0
	Regulation of protein cytokine secretion	5.2
	Regulation of phosphorylation	4.8
	Response to molecule of bacterial origin	3.8
*Mah* 1655 compared to uninfected	Inflammatory response	15.5
	Negative regulation of apoptosis	9.8
	Induction of apoptosis	6.6
	Cytokine/chemokine activity	5.9
	Regulation of T and B cell activation/proliferation	4.5
	Regulation of phosphorylation	4.2
	Response to molecule of bacterial origin	4.1
	Regulation of viral replication	3.6
	Pattern recognition receptor pathway signalling	3.5
	Cell migration	3.5
*Maa*1794 compared to uninfected	Inflammatory response	16.5
	Negative regulation of apoptosis	14.2
	Induction of apoptosis	13.0
	Response to molecule of bacterial origin	8.3
	Regulation of signal transduction	8.2
	Cytokine/chemokine activity	7.6
	Regulation of T and B cell activation/proliferation	6.6
	Regulation of cytokine production	6.0
	Regulation of protein cytokine secretion	4.9
	Regulation of phosphorylation	4.3

The analysis was done using the Database for Annotation, Visualization and Integrated Discovery (DAVID). An Enrichment score > 1.3 was considered statistically significant.

**Table 3:** Canonical pathway analyses

	**Up-regulated**	**p-value**
*Mah* VI101 compared to uninfected	TNFR2 signaling	6.3E-14
	Role of macrophages, fibroblasts and endothelial cells in rheumatoid arthritis	5.0E-11
	Death receptor signaling	5.0E-11
	Regulation of cytokine production in macrophages and Th cells by IL-17A and IL-17 F	6.3E-11
	Role of IL-17A in arthritis	1.9E-10
	TNFR1 signaling	2.8E-10
	TREM1 signaling	7.6E-10
	Regulation of cytokine production in intestinal epithelial cells by IL-17A and IL-17 F	1.4E-09
	Hepatic fibrosis/Hepatic stellate cell activation	1.7E-09
	IL-6 signaling	1.9E-09
*Mah* 1655 compared to uninfected	TNFR2 signaling	6.3E-12
	4-1BB signaling in T lymphocytes	7.2E-10
	TNFR1 signaling	1.1E-09
	Small cell lung cancer signaling	2.9E-09
	Role of IL-17A in arthritis	4.0E-09
	Death receptor signaling	7.6E-09
	Induction of apoptosis by HIV1	8.9E-09
	iNOS signaling	1.4E-08
	Type I diabetes mellitus signaling	1.5E-08
	Regulation of cytokine production in macrophages and Th cells by IL-17A and IL-17 F	1.9E-08
*Maa*1794 compared to uninfected	Death receptor signaling	1.0E-13
	TNFR2 signaling	1.0E-12
	Role of macrophages, fibroblasts and endothelial cells in rheumatoid arthritis	2.5E-11
	Small cell lung cancer signaling	6.3E-11
	Role of IL-17 F in allergic inflammatory airway diseases	1.4E-10
	TWEAK signaling	3.0E-10
	Role of IL-17A in arthritis	3.7E-10
	TNFR1 signaling	4.1E-10
	Regulation of cytokine production in macrophages and Th cells by IL-17A and IL-17 F	5.1E-10
	IL-17A Signaling in fibroblasts	7.1E-10
	**Down regulated**	**p-value**
*Mah* VI101 compared to uninfected	Role of CHK proteins in cell cycle checkpoint control	1.2E-04
	Tetrapyrrole biosynthesis II	1.9E-04
	Galactose degradation I (Leloir pathway)	1.9E-04
	Mismatch repair in Eukaryotes	7.8E-04
	Heparan sulfate biosynthesis	8.1E-04
	Role of BRCA1 in DNA damage response	1.2E-03
	Heme biosynthesis II	1.5E-03
	Glycine betaine degradation	2.1E-03
	Coenzyme A biosynthesis	2.2E-03
	L-serine degradation	2.2E-03
*Mah* 1655 compared to uninfected	Colorectal cancer metastasis signaling	4.4E-04
	Hereditary breast cancer signaling	1.3E-03
	Role of BRCA1 in DNA damage response	1.9E-03
	IL-12 signaling and production in macrophages	2.2E-03
	Sulfite oxidation IV	4.2E-03
	Antiproliferative role of TOB in T cell signaling	5.1E-03
	Dendritic cell maturation	6.0E-03
	Role of NFAT in cardiac hypertrophy	6.5E-03
	HMGB1 signaling	6.6E-03
	Superpathway of inositol phosphate compounds	6.9E-03
*Maa*1794 compared to uninfected	Mismatch repair in Eukaryotes	6.3E-07
	Role of BRCA1 in DNA damage response	2.9E-06
	CHK proteins in cell cycle checkpoint control	5.6E-06
	CTLA4 signaling in cytotoxic T lymphocytes	7.9E-06
	Hereditary breast cancer signaling	1.5E-05
	Growth hormone signaling	1.7E-05
	Molecular mechanisms of cancer	1.7E-05
	ERK5 signaling	2.7E-05
	Production of NO amd ROS in macrophages	3.8E-05
	B cell receptor signaling	1.3E-04

Pathways induced by infection of human monocyte-derived macrophages with *M. avium* isolates.

The analysis was done in Ingenuity Systems software.

The ten top pathways are shown.

**Table 4:** Upstream regulator analyses

	**Upstream regulator**	**Activation z-score**	**p-value of overlap**
*Mah* VI101 compared to uninfected	TNF	12.15	1.40E-76
	NFkB (complex)	9.02	1.14E-60
	IL1B	9.32	9.77E-58
	CD40LG	6.34	2.80E-56
	TREM1	2.56	2.45E-49
	IFNG	7.68	2.73E-42
	RELA	6.59	3.37E-42
	TNFSF11	6.16	5.99E-41
	NFKBIA	4.12	8.41E-40
	IKBKB	5.41	2.25E-35
*Mah* 1655 compared to uninfected	TNF	8.45	2.79E-58
	NFkB (complex)	6.45	1.57E-42
	CD40LG	4.03	5.71E-39
	IL1B	6.52	8.67E-39
	IL1A	4.97	1.08E-27
	TNFSF11	4.30	1.12E-27
	RELA	5.07	4.74E-27
	IFNG	5.50	1.72E-26
	NFKBIA	2.86	2.75E-26
	TREM1	2.18	2.80E-26
*Maa*1794 compared to uninfected	TNF	12.36	6.80E-70
	NFkB (complex)	9.52	6.86E-63
	CD40LG	6.40	2.02E-55
	IL1B	9.70	7.41E-52
	IFNG	8.72	8.88E-44
	NFKBIA	3.01	1.96E-39
	RELA	6.92	2.09E-37
	TNFSF11	6.55	3.60E-37
	TLR4	6.58	5.10E-36
	IL1A	7.19	7.30E-36

Genes predicted to act as up-stream regulators for the observed responses after infection of human monocyte-derived macrophages with *M. avium* isolates.

The analysis was done in Ingenuity Systems software. Responses to chemicals/drugs are excluded. The ten top genes are shown.

**Figure 4:**
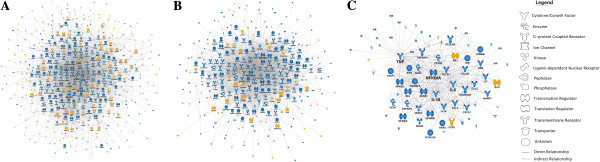
**Gene networks triggered in human macrophages infected with different isolates of *Mycobacterium avium*.** Cells have been incubated with *M. avium* subsp. *avium* 1794 **(A)**, *M. avium* subsp. *hominissuis* VI101 **(B)** and *M. avium* subsp. *hominissuis* 1655 **(C)** for four hours. Blue hubs indicate up-regulation, yellow indicate down-regulation of the respective gene. The size of the gene name reflects the number of links to other genes in the network, and genes with < 5 genes are minimized. For this analysis the number of genes was reduced, in order to comply with the limitation of the IPA software, by increasing the stringency of the statistical analysis to FDR < 0.01 from FDR < 0.05.

To identify the putative molecular mechanisms that are regulating the responses, we constructed molecular interaction networks for each isolate using mechanistic findings from previous studies. This analysis showed that *Maa* 1794 induced the largest gene network in human macrophages, followed by *Mah* VI101, then by *Mah* 1655 (Figure 4). The networks were organized around a series of hyper-connected hubs, and the most important hubs (based on number of interactions) are listed in the Table 5. Notably, the network connectivity patterns were dominated by TNF, IL-1B, IL-6, NFkB1, NFkBIA, PTGS2 and others, and many of these pathways were also highlighted by the upstream regulator analysis.

**Table 5:** **Top 20 hub genes and number of connections induced by infection of human monocyte-derived macrophages with *M. avium *isolates**

***Mah *1655**		***Mah *V101**		***Maa *1794**	
**Gene ID**	**No. of interactions**	**Gene ID**	**No. of interactions**	**Gene ID**	**No. of interactions**
TNF	61	TNF	178	TNF	219
IL1B	43	IL6	117	IL6	141
IL6	38	IL1B	113	IL1B	127
NFKBIA	34	NFKBIA	81	NFKBIA	93
NFKB1	27	IL10	80	FOS	88
IL1A	22	NFKB1	70	NFKB1	76
PTGS2	19	FOS	69	CSF2	66
NFKB2	18	CSF2	59	ILIA	63
CXCL2	18	ILIA	58	BCL2	59
CCL5	18	JUN	57	OSM	59
OSM	17	CD40	53	CD40	58
TNFAIP3	14	PTGS2	52	PTGS2	56
CCL3L1	14	Rb1	50	SRC	55
LIF	14	BCL2	47	RB1	50
CCL20	14	OSM	47	IL15	50
REL	13	VEGFA	46	CXCL2	45
SOD2	13	SRC	43	CCL5	42
NFKBIB	11	CCL5	42	PIK3R1	42
CCL3	11	CXCL2	42	CEBPA	41
TRAF1	10	NFKB2	38	TICAM1	41

### Differential gene expression elicited by *Maa* 1794, *Mah* VI101 and *Mah* 1655

At FDR < 0.05, no significant differences were observed between cells infected with *Maa* 1794 and *Mah* VI101, while only two genes (IL6, GPR109A) were upregulated in *Maa* 1794 compared to *Mah* 1655 and one gene (IL-6) was upregulated in *Mah* VI101 compared to *Mah* 1655.

### Protein analysis

Gene expression analysis pointed to apoptosis and immune response as two major biological functions upregulated in response to all three *M. avium* isolates, albeit at lower levels in *Mah* 1655 compared to *Maa* 1794 and *Mah* VI101. We thus attempted to look at proteins involved in apoptosis by western blot and cytokine by a multiplex assay. However, differences between the isolates with regards to apoptotic proteins were not detected, probably due to the lower sensitivity in this assay compared to the microarray analyses (data not shown).

**Figure 5:**
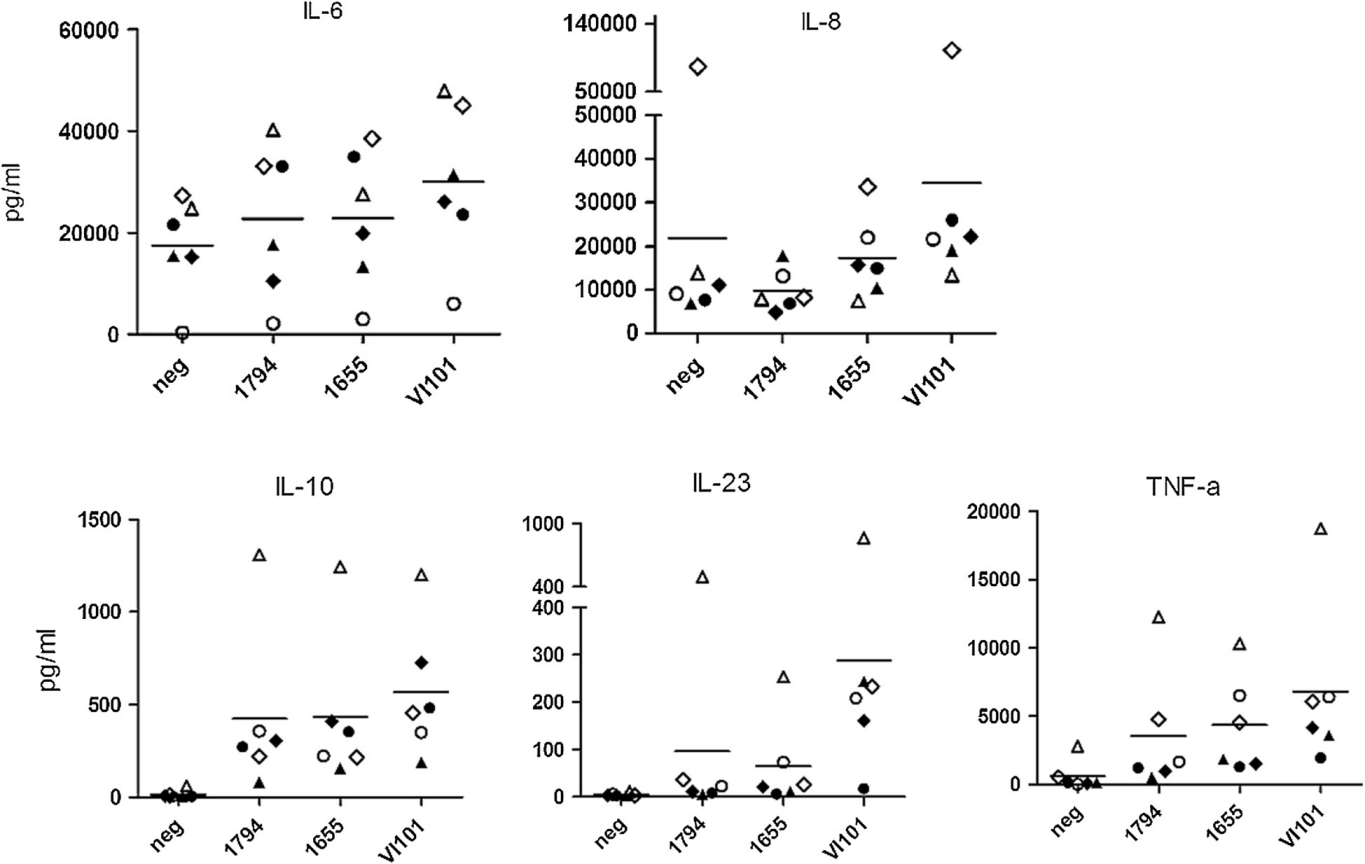
**Cytokine expression from primary monocyte-derived macrophages.** CD14+ cells were obtained from six donors and allowed to mature into macrophages by incubation for five days. The cells were then infected with the three isolates, *Maa* 1794, *Mah* 1655 and *Mah* VI101, at a MOI of 10:1 for 24 hours or left uninfected (neg). The supernatant was removed and assessed for IL-6, IL-8, IL-10, IL-23 and TNF-α by the Bio-plex assay. The six donors are represented with individual symbols. Each point is the median of triplicate wells. The horizontal line represents the mean response of the six donors. *Mah* VI101, consistently produced more IL-8, IL-23 and TNF-α than *Maa* 1794 and more IL-23 than *Mah* 1655. The difference was significant (p < 0.05) using the non-parametric Wilcoxon signed rank test.

Macrophages infected with all three isolates consistently produced more IL-10, IL-23 and TNF-α than uninfected controls (Figure 5). The production of IL-6 and IL-8 in uninfected cells was often high, and in some donors infection led to a decrease in cytokine production. The decrease in cytokine production was particularly evident for IL-8 after infection with *Maa* 1794, to a lesser extent by infection with *Mah* 1655 and rarely by *Mah* VI101 (Figure 5). There was a large donor to donor variation in the amount of cytokines produced, and this variation surpassed the difference induced by the isolates. Nevertheless, *Mah* VI101 consistently induced more IL-8, IL-23 and TNF-α than *Maa* 1794 (p < 0.05) and more IL-23 than *Mah* 1655 (p < 0.05).

## Discussion

The present study describes the uptake and intracellular growth of *Maa* and *Mah* isolates in human monocytes, and characterises genome-wide transcriptional responses elicited by these isolates in macrophages. *Mycobacterium avium* isolates of different genotypic characteristics were all able to enter and persist within human primary monocytes. Intracellular replication was only seen by *Maa* suggesting that the differences in the ability to survive and replicate in macrophages cannot explain why humans are more often infected with *Mah*. The gene expression program induced by infection with *Maa* and *Mah* isolates was broadly comparable in terms of the biological functions, pathways, and gene networks that were perturbed. However, the degree of perturbation varied considerably. The different level of expression led to an apparent discrepancy in the number of unique genes induced by the various isolates and the lack of differences when comparing the isolates with each other. Many of the genes significantly induced by *Maa*1794 were also induced at a lower level by the other isolates (Additional file 2: Figure S1). This intermediate expression was neither significantly different from uninfected cells nor from *Maa*1794. A larger samples size might have given more significant differences between the isolates since large donor to donor variation is present when using primary cell lines.

In general, responses to *Maa*1794 were excessive, and were characterized by increased expression of genes downstream of type I and II interferon signaling. Further studies will be required to determine if variations in these pathways account for the exaggerated responses and/or differences in prevalence. Similar pathways were induced in ruminant monocyte derived macrophages infected with the closely related *Mycobacterium avium* subspecies *paratuberculosis (Map)*[25-27], while a study using the murine cell line RAW264.7 showed that *Maa* and *Mah* induced higher levels of pro-inflammatory genes compared to *Map*[28]. Together with our data, these studies suggest that similar pathways were induced by all *M. avium* subspecies. A direct comparison of the level of macrophage activation is however challenging considering the variation in cell cultures and isolates used in the various studies.

Macrophage activation can be interpreted as an appropriate reaction to an invading pathogen or as a failed attempt of the mycobacteria to silence the host’s defences. *Maa* 1794 and *Mah* VI101 differed completely in their ability to replicate within host cells, although both isolates induced a strong activation of macrophages and innate immune responses on the transcriptional level. Analyses on the protein level revealed that the final synthesis of inflammatory cytokines were higher in response to *Mah* VI101 compared to *Maa* 1794. One could thus argue that the inflammatory response was sufficient to limit growth of *Mah* VI101, but not *Maa* 1794. The complexity of the outcome of an infection, and *in vitro* culture, was illustrated by the fact that despite a low activation of the innate immune response *Mah* 1655 did not proliferate intracellularly. The microarray data provided no clear explanation to the lower level of inflammatory cytokines induced by *Maa* 1794 on the protein level compared to *Mah* VI101. However, regulation of inflammation on the post-transcriptional level has been demonstrated to be important [29], and a down-regulation of immune responses to avoid immunopathology can occur in chronic infections. The presence of unidentified virulence factors in *Maa* 1794 more actively shutting down the innate responses cannot be excluded. The genomes of the three isolates did for instance differ in the presence of GPL genes, and the involvement of GPLs in *M. avium* colony morphology and of ssGPLs in pathogenicity is increasingly recognised [30-32].

The induction of pro-apoptotic genes, such as RIPK2, BID and tBID was seen after infection with all three isolates, however apoptotic pathways were affected to a lesser degree by *Mah* 1655 than by *Maa* 1794 and *Mah* VI101. The correlation between apoptosis and virulence of mycobacterial strains is debated. Inhibition of apoptosis has been described as a virulence factor in mycobacteria [33-35], but it has also been suggested that *M. avium* can use apoptosis of macrophages as a tool for spreading [36]. One of the other more pronounced differences between the isolates was the increased expression of genes involved in activation of T cells like CD25, CD40, CD274 and IL23 in *Maa* 1794 and *Mah* VI101. CD25, CD40 and CD274 are transmembrane surface proteins involved in activation of macrophages and T cells upon binding, while IL-23 is essential for the persistence and function of Th17 cells [37,38]. These findings suggest that differential activation of apoptotic pathways and/or activation of T cells may occur in humans after infection with the different isolates. The lower activation of macrophages by *Mah* 1655 may be a useful strategy to avoid the induction of an adaptive immune response in the host. This isolate was included because it harbours IS*Mpa1* and has a similar IS*1245* RFLP pattern to strains shown to be associated with virulence [24,39]. Further studies, including experimental infection models, could provide an answer to whether the “silencing approach” of *Mah* 1655 might be the most successful strategy.

Other possible explanations for the low prevalence of *Maa* observed in the human population, and also in pigs, include the lack of exposure or a reduced ability to cross the human intestinal barrier. In the Norwegian pig population only *Mah* has been isolated [24], but the virulence of *Maa* in pigs has been demonstrated by others [40], and previous research demonstrated that *Maa* 1794 and *Mah* VI101 were equally able to infect piglets [41]. It thus appears that a lack of exposure is the most likely explanation for the difference in prevalence in pigs. The virulence of *Maa* has also been demonstrated in mice [42]. Although the study of Pedrosa et al. was performed prior to the division of *M. avium* into subspecies, one could assume that the bird isolates used in the older studies are equivalent with *Maa*, and the human isolates with *Mah*. The virulence of *M. avium* subspecies in humans can obviously not be determined *in vivo*. However, altogether the studies in human primary cell lines and infection studies in other species, suggest that different exposure to *Mah* and *Maa* in humans is a plausible explanation for the difference in prevalence.

## Conclusion

In conclusion, our findings demonstrate that the ability to replicate in macrophages cannot explain the difference in prevalence of the two subspecies in humans. Genome-wide expression profiling studies further showed that similar biological pathways and networks were elicited by the two subspecies, although responses to the *Maa* subspecies were exaggerated.

### Availability of supporting data

The raw microarray data have been made publicly available in the Array Express repository http://www.ebi.ac.uk/arrayexpress/experiments/E-MTAB-1101/. The supporting data are included in the additional file.

## Competing interests

The authors state that there are no competing interests related to the present study.

## Authors’ contributions

AA was responsible for conception and design of the experiments, contributed to work on immunoblotting, performed work with cell-culture and bacterial quantification, data analysis and drafted the manuscript. AJ contributed to analysis of microarray data and critical revision of the manuscript. AF contributed to microarray analysis and critical revision of the manuscript. TBJ contributed to conception and design of the experiment and critical revision of the manuscript. IAH contributed to cell-culture, immunoblotting and critical revision of the manuscript. BD contributed to conception and design and critical revision of the manuscript. AB contributed to analysis of microarray data and drafting of the manuscript. IO was involved in conception and design of the experiment, performed cytokine profiling and contributed to drafting of the manuscript. All authors read and approved the final manuscript.

## Supplementary Material

Additional file 1: Table S1 List of genes differentially expressed in strain *Maa* 1794, *Mah* VI101 and *Mah* 1655 compared to uninfected cells. Click here for file

Additional file 2: Figure S1 Volcano plots comparing gene expression patterns in human marophages cultured in the presence or absence of different isolates of *Mycobacterium avium.***A: ***Maa* 1794. **B: ***Mah* VI101. **C: ***Mah* 1655. In all three plots, the red data points show the location of genes that were upregulated in responses to isolate Maa 1794. Click here for file
