# Supplementary material for: Intracellular growth of Mycobacterium avium subspecies and global transcriptional responses in human macrophages after infection
Source: BMC Genomics. 2014 Jan 23;15:58. doi: 10.1186/1471-2164-15-58 (PMC3906092; doi:10.1186/1471-2164-15-58)
Supplement: Additional file 2: Figure S1 — Volcano plots comparing gene expression patterns in human marophages cultured in the presence or absence of different isolates of Mycobacterium avium.A: Maa 1794. B: Mah VI101. C: Mah 1655. In all three plots, the red data points show the location of genes that were upregulated in responses to isolate Maa 1794. [file 1471-2164-15-58-S2.pdf]

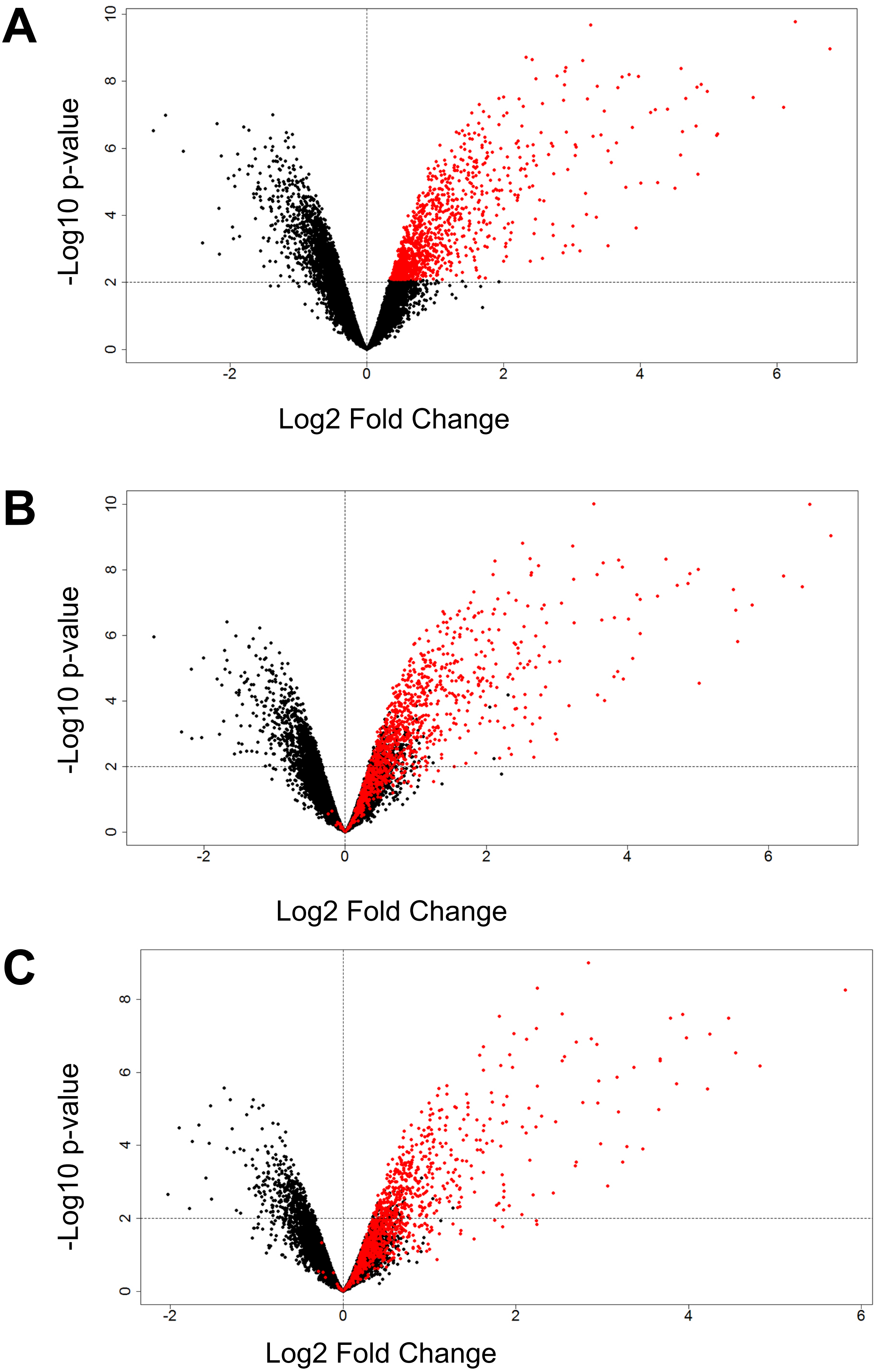

Figure S1: Volcano plots comparing gene expression patterns in human marophages cultured in the presence or absence of different isolates of *Mycobacterium avium.* **A:** Maa 1794. **B:** Mah VI101. **C:** Mah 1665. In all three plots, the red data points show the location of genes that were upregulated in responses to isolate Maa 1794.
